# Nasal mucosa study of leprosy contacts with positive serology for the phenolic glycolipid 1 antigen

**DOI:** 10.1590/S1808-86942010000500008

**Published:** 2015-10-22

**Authors:** Ana Cristina da Costa Martins, Alice Miranda, Maria Leide Wan-del-Rey de Oliveira, Samira Bührer-Sékula, Alejandra Martinez

**Affiliations:** 1 Doctoral degree, otorhinolaryngologist at the Fundação Oswaldo Cruz/FIOCRUZ- RJ; 2 Doctoral degree, Leprosy Laboratory - Instituto Oswaldo Cruz - IOC - FIOCRUZ, RJ; 3 Doctoral degree, graduate course in dermatology, Faculdade de Medicina, UFRJ, RJ; 4 Doctoral degree, Institute of Tropical Diseases and Public Health - Goiania, GO; 5 Doctoral, Department of Micobacterioses, Instituto Oswaldo Cruz, FIOCRUZ, RJ. Fundação Oswaldo Cruz - IOCRUZ Universidade Federal do Rio de Janeiro - FRJ Netherlands Leprosy Relief

**Keywords:** endoscopy, glycolipids, nasal mucosa, mycobacterium leprae, polymerase chain reaction.

## Abstract

**Abstract:**

Leprosy is a chronic infectious disease caused by Mycobacterium leprae. The disease more frequently affects the nasal mucosa and can occur independently of its clinical form or even before lesions on the skin or on other parts of the body. It is necessary to employ epidemiological surveillance of household contacts with new leprosy cases for early disease diagnosis.

**Aim:**

identify specific and early leprosy lesions through endoscopic, baciloscopy, histopathology exams, and real time polymerase chain reaction of the nasal cavity mucosa on household and peridomiciliary contacts with positive serology for the phenolic glycolipid 1 antigen.

**Methodology:**

Between 2003 at 2006 there was a prospective cross-sectional clinical study with 31 contacts with patients with leprosy with positive serology against PGL-1, 05 negative controls and 01 positive control.

**Results:**

Between seropositive contacts, real-time PCR was positive for M. leprae DNA in 06 (19.35%) of them and the higher number of genome copies were found in contacts who became sick.

**Conclusion:**

Nasal mucosa tests alone did not enable the early diagnosis of Leprosy. However, through the combination of various methods, tests on the contacts can help identify subclinical infection and monitor the contacts that could be responsible for spreading the disease.

## INTRODUCTION

Rabello classified leprosy as a polar disease with two forms (tuberculoid leprosy or TT, and lepromatous leprosy or LL) based on the bodily response to this infection starting with an initial indeterminate form (indeterminate leprosy or IL). New clinical forms - borderline forms - were added to this classification in the VI International Leprosy Conference of 1953 in Madrid. These new dimorphic forms were attributed to patients that progressed from IL to uncharacteristic clinical presentations of polar TT (paucibacillary with high cellular immune response) and LL (multibacillary and low cellular immune response).

After the introduction of polychemotherapy (PCT/ WHO) in the 1980s, new diagnostic tools for an early diagnosis of leprosy have been sought. The ability to identify groups at an increased risk in highly endemic areas in and out of households, together with BCG vaccination of contacts, are measures developed in Brazil to help decrease the multibacillary forms of this disease. The efficacy of these measures, however, has been compromised by several operating issues that have resulted in high detection coefficients in the majority of Brazilian states.

Disease transmission has been debated for year; the upper airways, in particular the nose, appear to be the main entry and transmission route for Mycobacterium leprae. It is thought that 95% of LL patients will have an early involvement of the nose. There are specific histopathological changes in the mucosa even without visible lesions.^1^ There are many mucus-producing cells, edema, and increased vascularization of the plasmocyte and lymphocyte-infiltrated submucosa in the bacillary invasion phase of LL patients. This significant amount of mucus explains the typical nasal block and rhinorrhea in this initial stage. A proliferation phase ensues, in which these findings are exacerbated, resulting in a granulous aspect of the mucosa; at this point, macrophages predominate in the inflammatory infiltrate. In the next stage the mucosa becomes ulcerated and damaged; inflammation consists of macrophages and numerous bacilli, lymphocytes and plasmacytes. In the final phase - resolution and fibrosis - bacilli are rare and fibrosis is intense.^2^

The nasal epithelium is ciliated cylindrical pseudostratified with goblet cells, and rarely remains normal because of multiple insults. Such insulting factors include: extreme temperatures, infection, pollution, and trauma. This continuous aggression decreases the number of cilia on which air interacts, and increases the number of goblet and inflammatory cells. The progression of squamous metaplasia in this context starts in childhood; it is a normal phenomenon, a protective response to external factors. It is often seen in allergic rhinopathy.^3^

Histopathology is extremely valuable for diagnosing and classifying the clinical forms of leprosy, especially in indeterminate cases; this approach may show early on to which polar type (tuberculoid or lepromatous) the disease will progress. Biopsies should be preserved in 10% formaldehyde or Millonig (buffered formaldehyde) and hematoxylin-eosin, Ziehl-Wade-Klingmuller or Fite Faraco stained.

Among the new support tools for an early diagnosis and prediction of these groups there is the serology test that detects antibodies against the specific phenolic glycolipid antigen 1 (PGL-1) of M. leprae. The PGL-1 is specific to this bacillus and comprises about 2% of the total bacterial mass; it is found in tissues, circulating blood and urine of multibacillary patients. This test has been used for diagnosis worldwide,^4^,^5^,^6^ and positivity is proportional with the bacillary load; increased exposure to bacilli relates with a higher test positivity, ranging from 1+ (low bacillary load) to 4+ (high bacillary load). The DNA-amplification method using the polymerase chain reaction (PCR) technique appears to be more specific and sensitive for detecting bacilli in the nasal mucosa.^7^, ^8^, ^9^, ^10^, ^11^, ^12^, ^13^, ^14^, ^15^, ^16^, ^17^, ^18^ Several cohort studies,^9^,^10^,^14^, ^15^, ^16^, ^17^ based on serum positivity (anti-PGL-1) and PCR investigation of bacilli in the nasal mucosa, have shown persistent subclinical infection, especially in highly endemic areas.

These numbers guided an endoscopic study of the nasal cavity mucosa for investigating subclinical infection in serum positive contacts in an urban area of metropolitan Rio de Janeiro, the Duque de Caxias municipality. At the beginning of the study in 2003, the incidence and the prevalence of leprosy were respectively 5.04 and 7.29 per 10 thousand inhabitants in the 2nd district - the study micro area. A high endemic rate is evidenced by the fact that 11% of new cases were subjects aged below 15 years, which reflects active and recent transmission of this disease, as shown in several studies.^19^, ^20^, ^21^, ^22^ In 2003 there was a high rate of new cases with deformity, especially in the 1st - and most populated - district (10.7%), 75.7% of the cases that were evaluated.

## METHOD

A cross-sectional study was carried out from 2003 to 2006 of 31 contacts of leprosy patients that were positive for PGL-1 (out of 1886 contacts that comprised the total sample in the serological investigation),^23^ and 6 controls, of which one was positive and 5 were negative. Contacts and controls underwent nasal endoscopy, nasal mucosa smears, and lower right turbinate biopsy for acidfast bacilli (AFB) testing, histopathology and real-time polymerase chain reaction (RT-PCR). A new data base was built (using SPSS for Windows) containing the following data: sex, age, result of anti-PGL-1, the clinical form of the index case (MB-multibacillary or PB-paucibacillary), the type of relationship (relatives or social relations) with the index case, the type of contact (household or peridomiciliary) with the index case, the type of exposure (daily, weekly or every two weeks) with the index case, otorhinolaryngological complaints, nasal endoscopy, AFB, histopathology, and RT-PCR.

The study project was approved - see MEMO-no. 146/03 and MEMO-no. 0015.0.009.000-04. After accepting and signing the free informed consent form, positive test contacts aged over 15 years underwent a clinical otorhinolaryngological investigation; all received care according to bio-safety guidelines.

## RESULTS

Table 1. Serology and sex, age, index case classification, type of relation, type of contact, type of exposure to the index case, and RT-PCR; the confidence interval (CI) was 95%.

**Table 1:** Relation of serology with sex, age, classification of the index case, type of family relation, type of contact with the index case, and RT-PCR, with a 95% confidence interval (CI).

	2	+ (n = 24)		3+ (n = 6)		4+ (n = 1)	Total	p
Variables	n^o^	%	n^o^	%	n^o^	%	%	
male	12	70,6	5	29,4			100	0,182
female	12	85,7	1	7,1	1	7,1	100	0,182
> 32	9	64,3	4	28,6	1	7,1	100	0,234
< 32	15	88,2	2	11,8			100	0,234
CI-MB	18	75	5	20,8	1	4,2	100	0,782
CI-PB	6	85,7	1	14,3			100	0,782
household	16	66,7	1	16,7	1	100	58,1	0,059
peri-domiciliary	8	33,3	5	83,3			41,9	0,059
daily	18	75	5	20,8	1	4,2	100	0,197
+1x /week			1	100			100	0,197
1x /week	6	100					100	0,197
1^st^ degree	11	91,7			1	8,3	100	0,069
2^nd^ degree	7	58,3	5	41,7			100	0,069
3^rd^ degree	3	75,0	1	25			100	0,069
4^th^ degree	3	100					100	0,069
RT-PCR pos.	5	83,3			1	16,7	100	0,060
RT-PCR neg.	19	76,0	6	24			100	0,060

**Key:** n = total; > 32 - age over 32 years; < 32 - age below 32 years; IC = index case of contact; 1st degree relative = parents, children, siblings; 2nd degree relatives = uncles/aunts, cousins, grandparents, grandchildren, nephews/nieces; 3rd degree relatives = spouse, son-in-law, daughterin-law, stepfather/stepmother, stepchildren; 4th degree relative = friend, boy/girlfriend, tenants; RT-PCR pos. = positive real time PCR; RT-PCR neg. = negative real time PCR; p= p value.

### Nasal endoscopy

**Figure 1:**
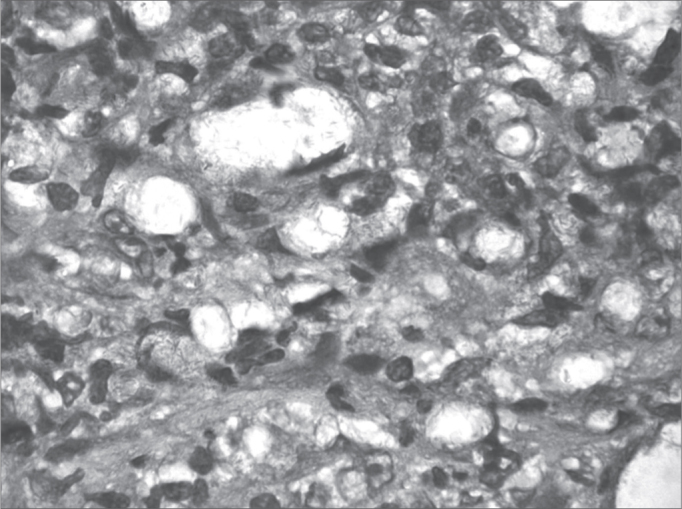
Slide at 1000x magnification, WADE stained - leprous rhinitis with vacuolated macrophages and bacilli (in red) in clumps.

**Figure 2:**
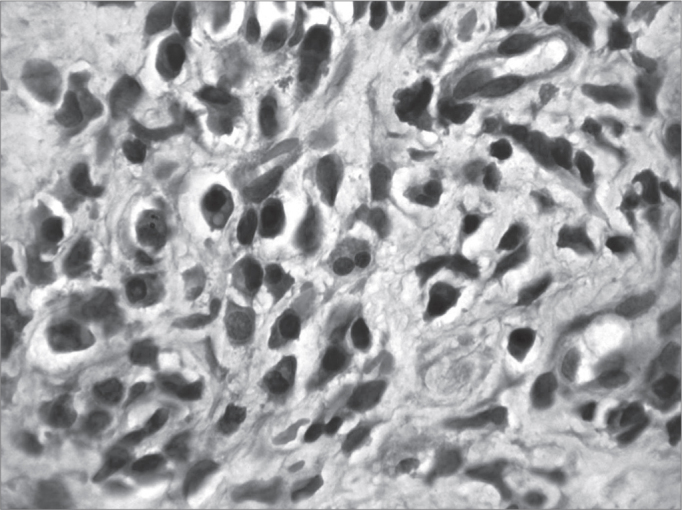
Acute over chronic rhinitis - slide at 1000x magnification, hematoxylin-eosin stained - slide showing eosinophils (binucleated cell in the center)

**Figure 3:**
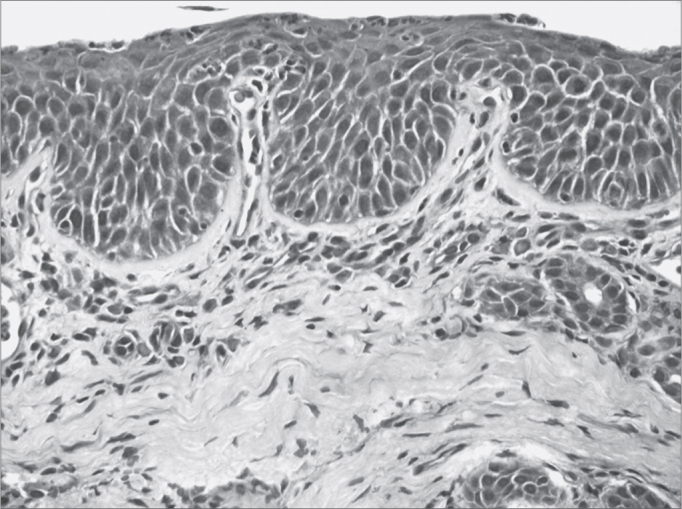
Moderate chronic rhinitis - slide at 400x magnification, hematoxylin-eosin stained - slide showing squamous metaplasia (well defined darker pink area on the surface)

**Figure 4:**
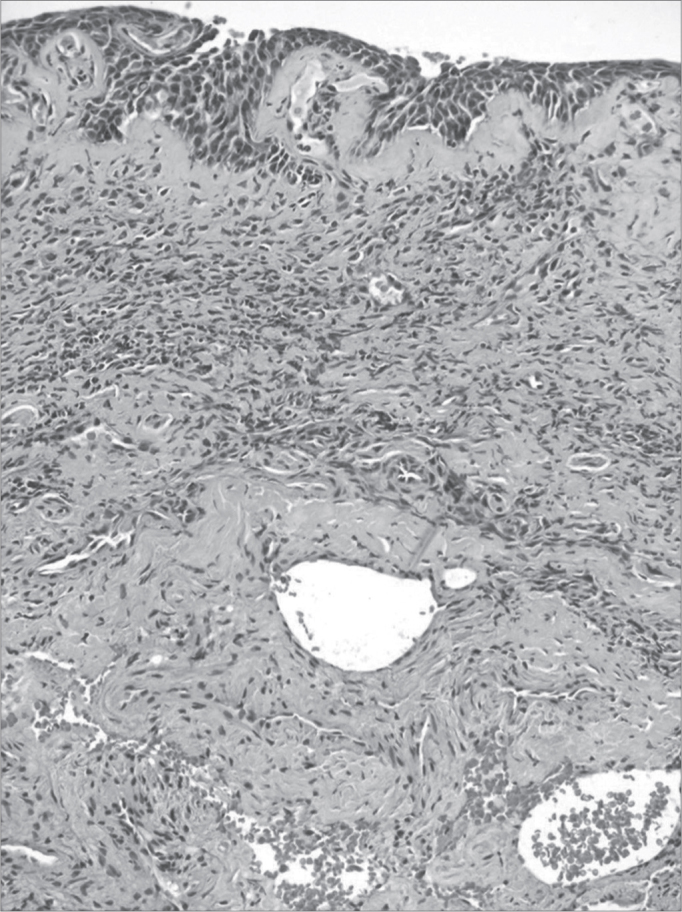
Severe chronic rhinitis - slide at 400x, hematoxylin-eosin stained - slide shows an intense inflammatory infiltrate with areas of bleeding

**Figure 5:**
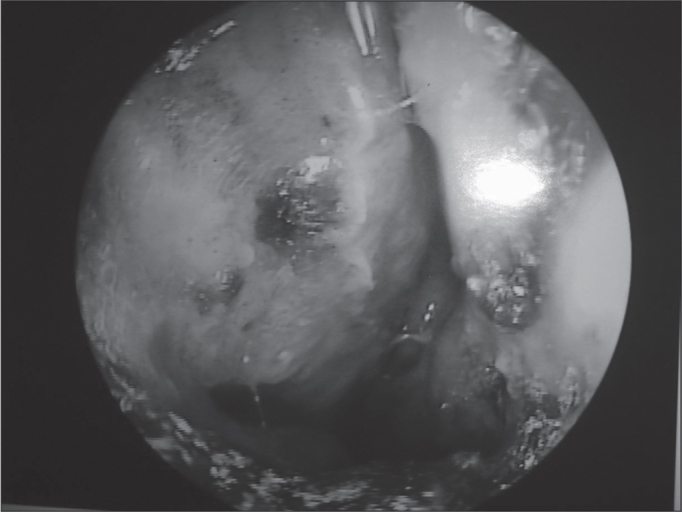
Leprous rhinitis - nasal endoscopy of the positive control showing a diffuse infiltration of the mucosa, blood crusts and dry mucosa to the left.

**Figure 6:**
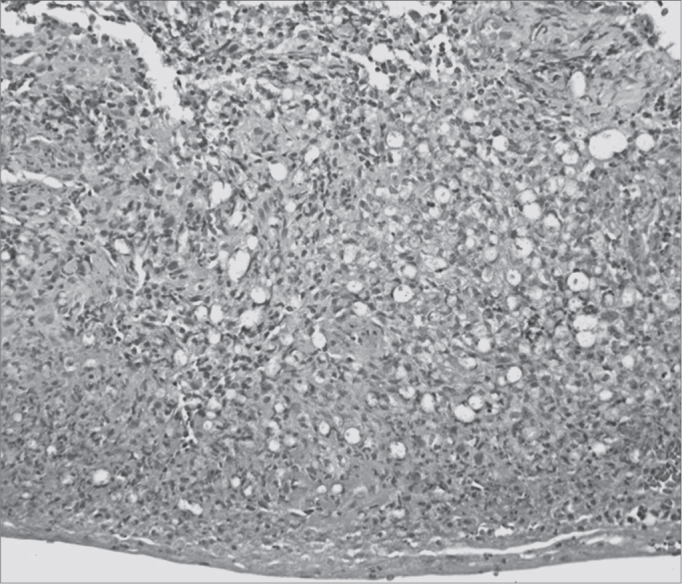
Leprous rhinitis - slide at 200x magnification, hematoxylin-eosin stained - dense inflammation (cells with purple nuclei) and many vacuolated macrophages.

**Figure 7:**
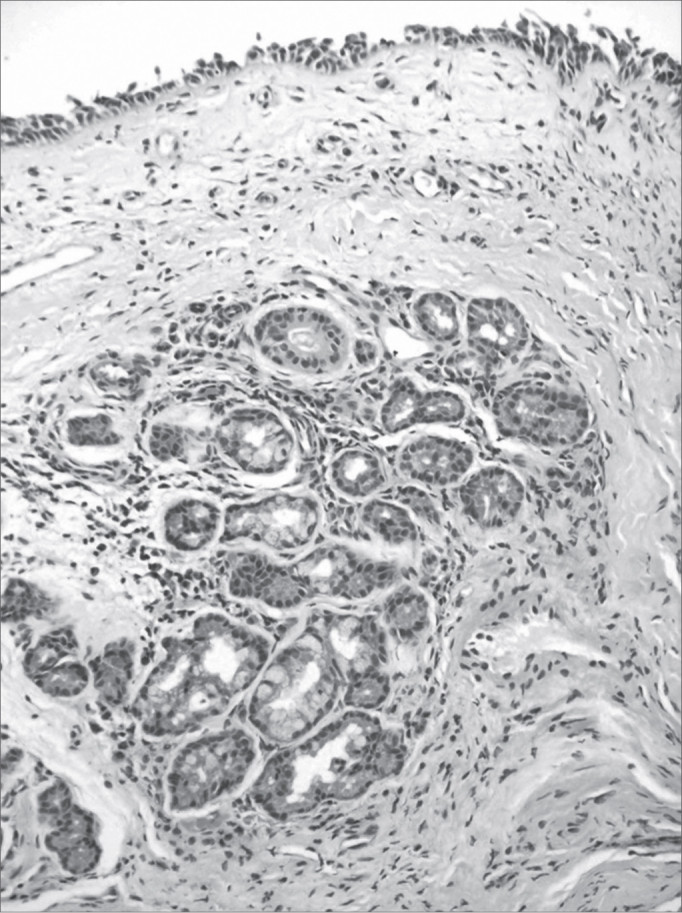
Mild chronic rhinitis - slide at 200x magnification, hematoxylin-eosin stained - slide showing a mild inflammatory infiltrate (cells with purple nuclei and clear pink tissue) and gland dilatation (central nodular aspect).

Figure 1, Figure 2, Figure 3, Figure 4, Figure 5, Figure 6, Figure 7. LEPROUS RHINITIS - Nasal endoscopy of a positive control: typical nasal mucosa in leprous rhinitis showing diffuse infiltration of the mucosa, dry mucosa, superficial ulcers, blood crusts, ectasia, and areas of bleeding.

### Histology

Semiquantitative analysis of histopathology findings in contacts was carried out; these were not specific for leprosy, and were classified as allergic rhinitis of varying intensity. Histopathology was carried out of the positive control (leprous rhinitis).

### Slides

Illustration 1: WADE-positive control / leprous rhinitis
Illustration 2: RCA/ HE (contact number 19, who became ill)
Illustration 3: RCM/HE
Illustration 4: RCS / HE
Illustration 6: LEPROUS RHINITIS / HE
Illustration 7: RCL / HE

### Results of laboratory tests in this study

There were 6 contacts with positive serology and positive DNA amplification (RT-PCR). The index cases in 4 of these contacts were multibacillary patients; in another 2 contacts, the index cases were paucibacillary. Five among the 6 contacts had positive 2+ serum levels and one had a 4+ serum level. The age ranged from 16 to 70 years; there were 4 females and 2 males.

## DISCUSSION

The study sample is small compared to the whole series - 1886 contacts - not only because negative, doubtful and positive 1+ anti-PGL-1 cases were excluded, but also because subjects raised several hurdles: resistance from asymptomatic individuals, distance and transportation difficulties, subjects of lower social and economic status, and the possibility of undergoing invasive procedures such as a nasal biopsy. Thus, for safety and operational aspects, the study focused on subjects aged over 15 years. The serological inquiry revealed that 31% of positive contacts were aged less than 15 years, and were therefore excluded.

It is important for epidemiological purposes to investigate contacts outside households. It is possible to identify transmission sources and subclinical infection in peri-domiciliary contacts, and to understand the true role of the nasal mucosa in this context. The percentages among serum positive cases were 58.1% for household cases and 41.9% for peri-domiciliary cases. Most of the latter cases were 3+ serum positive (83.3%), and most of the former cases were 2+ serum positive (66.7%). Coexisting in a common area with index cases is habitual in the Duque de Caxias municipality, which thus characterizes the concept of a peri-domiciliary space. Daily closeness to 1st and 2nd degree relatives was more evident in 2+ serum positive cases; for 3+ contacts the most common family relation was 2nd degree relatives (Table 1). The possibility of subclinical infection is raised based on the results, as extrapolation of household limits was significant in the serological context (p = 0.059 / Table 1).

There were no significant percentage correlations between contacts with positive sera and the clinical form of the index case; nevertheless, there was a tendency for a higher rate of mutibacillary index cases. The index case clinical form was unrelated to serum positivity of contacts; this index case was not the primary case or the source of transmission for the secondary case.^23^

A comparison of serology (anti-PGL-1) and RT-PCR results showed that the percentages are close to statistical significance (p=0.060/Table 1). This underlines the importance of exposure to bacilli and its effect on serology, even in contacts with lower serum levels. However, the clinical form of the index case was unrelated with PCR positivity. Although the multibacillary clinical form predominated, there was no statistically significant difference compared with contacts exposed to the paucibacillary clinical form. This result underlines the importance of including the paucibacillary form as a disease transmission source. Van Beers^12^ and Moet^24^ have suggested that contacts of paucibacillary patients appear to be at a higher risk of becoming infected compared to the general population.

In the context of transmission, we found 7 contacts of paucibacillary index cases. Of these, 2 had positive serology and RT-PCR, and 5 had positive serology only. Of the former 2, serology increased from 2+ to 4+ in one patient after investigation of the mucosa, and the patient eventually became ill; the second patient remained unaltered. The contact that became a case had no history of any contact with multibacillary cases; thus, there are doubts about the true source of transmission. This finding is important in the epidemiological chain of events in the disease when taking into account multibacillary patients as the only disease transmission source. As in most studies,^9^,^10^, ^12^,^14^, ^15^, ^16^, ^17^,^25^, ^26^, ^27^, ^28^, there were 24 contacts of multibacillary index cases.

Note that the detection of anti-PGL-1 antibodies may support disease classification because it expresses bacillary load, supports the diagnosis and may help identify infected contacts.^5^,^6^,^29^ Serology, however, do not necessarily mean infection; in most people, it expresses resistance to M. leprae, and if such individuals become ill, they have a low bacillary load (paucibacillary) and therefore low anti-PGL-1 IgM immunoglobulin levels.^4^,^29^, ^30^, ^31^

It is assumed that contacts have transient subclinical infection - with or without the disease progressing - as a consequence of the nasal discharge of bacilliferous patients. Thus, it has been suggested that the nasal mucosa is the probable site of an initial immune response.^9^,^10^,^13^,^15^,^17^,^26^,^32^, ^33^, ^34^, ^35^, ^36^, ^37^, ^38^, ^39^ Some authors have postulated that leprosy patients as well as healthy contacts may be asymptomatic carriers of M. leprae in their nasal mucosa.

Note that most contacts reported non-specific complaints reminiscent of allergic rhinopathy, such as nasal block (35.5%), pruritis (25.8%), rhinorrhea (29%) and sneezing (9.7%). Added to these complaints are humid and poorly ventilated households, and dusty and unpaved roads, favorable environments for M. leprae. It sits on the nasal mucosa and during the initial phase of the disease may cause nasal block and rhinorrhea, which are often mistaken for a common cold. Still, no association between the presence of otorhinolaryngological complaints and positive RT-PCR tests has been found (p=0.318).

Constant aggression from the environment may explain the histology of contacts. In the contact that became a disease case a year after the nasal mucosa test (Frame 1, Frame 2, Frame 3), the nasal mucosa histology led to a classification of acute episodes in chronic allergic rhinitis. This finding could support the hypothesis of leprous rhinitis occurring at the initial phase when bacilli are deposited on the nasal mucosa.^2^ At this point, classical symptoms - nasal block and rhinorrhea - would ensue. This analysis is semi-quantitative, and superior results could be gained with morphometric histological studies (quantitative) of the mucosa of contacts; this requires further study.

**Frame 1:** Results of laboratory tests of leprosy contacts.

N	male	female	CI -PB	CI-MB	PGL1 2+	PGL1 3+	PGL1 4+	BAAR	PCR-RT	RCL	RCA	RCM	RCI
31	15	16	07	24	24	06	01	0	06	09	09	09	03

**key:** N (total no. of contacts); IC-PB- number of paucibacillary index cases of contact; IC-MB - number of multibacillary index cases of contact, PGL -1 - number of contacts with positive serology (Ml Flow); EX. AFB MN - bacilloscopic exam of the nasal mucosa; Real time PCR - number of contacts tested positive, Histopathology: RCL - mild chronic rhinitis; RCM - moderate chronic rhinitis; RCI - severe chronic rhinitis; RCA - acute chronic rhinitis.

**Frame 2:** Results of laboratory tests of the positive control.

N	SEX	AGE	CI	EX. BAAR MN	Anti-PGL-1	PCR	HISTOPATHOLOGY
1	M	26	NC	NEG	2+	POS	Vacuolated macrophages with numerous bacilli in clumps and intense inflammation

**key:** male patient, 26 years, negative nasal mucosa AFB test, unknown index case (IC), 2+ anti-PGL-1, positive PCR, and histopathology of leprous rhinitis.

**Frame 3:** Results of contacts with positive RT-PCR and contact that became ill.

Age	CI	Anti-PGL-1	PCR	Ct	Log of mass	mass	Genome copies
70	MB	4+	Pos	35,27	0,782178218	0,165128	41,28210089
40	PB	2+	Pos	35,62	0,897689769	0,126564	31,64100284
37	MB	2+	Pos	35,34	0,805280528	0,156574	39,14348425
30	PB	2+	Pos	28	1,6117161716	41,41539	10353,84657
21	MB	2+	Pos	35,02	0,699669967	0,199678	49,91947873
16	MB	2+	Pos	35,11	0,729372937	0,186478	46,61944204

**key:** Ct - number of cycles in RT-PCR; Log of mass

DNA mass expressed as log; mass DNA mass of M. leprae and number of genome copies. - contact that became ill:

Contact with the PB index case, 2+ PGL-1, with larger mass, more genome copies and lower Ct.

New techniques, such as RT-PCR, have improved the detection of M. leprae and supported traditional methods for diagnosing leprosy (bacilloscopy and histopathology); these are major advances compared to the clinical diagnosis only. Quantitative tests, which are more precise, may be carried out. As leprosy may be related to genetic and environmental factors, and exposure to bacilli, positive PCR testing in blood and nasal secretion samples of contacts does not necessarily characterize illness.16 Nasal mucosa biopsies and DNA amplification by RT-PCR revealed a 19.35% positivity rate for the presence of M. leprae among the sample contacts of this study. A comparison of our results with other studies in which the PCR technique was used (9.8%;9 7.8%;10 12.8%;14 4.6%;^15^ 1.7%;16 and 10.1%17), we found that the tested specimens varied (mucus swab or mucosa) as did PCR testing (conventional method in agarose gel plates), which may affect the results. Our study sample was smaller (n = 31), which may have increased the positivity rate among seropositive contacts. Still, the percentage (19.35%) was due to mucosa biopsy samples and RT-PCR, which is highly specific and sensitive, and thus adopted as the standard at the laboratory where these samples were analyzed. Further studies on this topic are recommended, with larger samples for improved analyses.

Absence of positivity in negative controls was evidence of the specificity and sensitivity of RT-PCR in positive contacts. The PCR identifies M. leprae and also makes it possible to quantify the DNA to help clarify the diagnosis of suspected cases. The contact that became ill in the year after the nasal mucosa test had a large number of M. leprae genome copies, and eventually serum conversion progressed from 2+ to 4+. Ramaprasad^35^ has suggested that such serological changes occur after infection of the nasal mucosa (PCR +) and eventual systemic dissemination and an immune response. Based on DNA quantification of contacts at a higher risk in studies with larger sample sizes and longer follow-up, it may be possible to establish a disease-indicating value.

Finding M. leprae in nasal cavities is relatively common in highly endemic areas. Nevertheless, PCR positivity does not differ between contacts and non-contacts, as mentioned above. It is thought that bacilli do not remain continuously on the nasal mucosa, and that most subclinical infections resolve spontaneously, never progressing to a disease. New techniques, such as serology, and molecular biology, have increased our knowledge of the epidemiology of leprosy; there are still important gaps in our knowledge of how this disease is transmitted.

## CONCLUSION

Nasal mucosa tests alone are insufficient for an early diagnosis of leprosy. However, by combining several methods and by examining contacts, subclinical infection may be identified, and patients at a higher risk - such as the contact that became ill - may be monitored.

No contact in our series had endoscopic or histological findings specific to leprosy; neither was the mucosa positive for AFB to make an early diagnosis possible.

## ACKNOWLEDGEMENT

Support from the Netherlands Leprosy Relief, SMSDC, FIOCRUZ and students from PINC - UFRJ.
